# Supplementary material for: The interactome of intact mitochondria by cross-linking mass spectrometry provides evidence for coexisting respiratory supercomplexes
Source: Mol Cell Proteomics. 2017 Dec 8;17(2):216–32. doi: 10.1074/mcp.RA117.000470 (PMC5795388; doi:10.1074/mcp.RA117.000470)
Supplement: Supplemental Data [file supp_RA117.000470_133922_0_supp_23978_fzffwf.zip › spectra_annotation/mito_spectra_annotation/312-1-4-1-7-1.pdf]

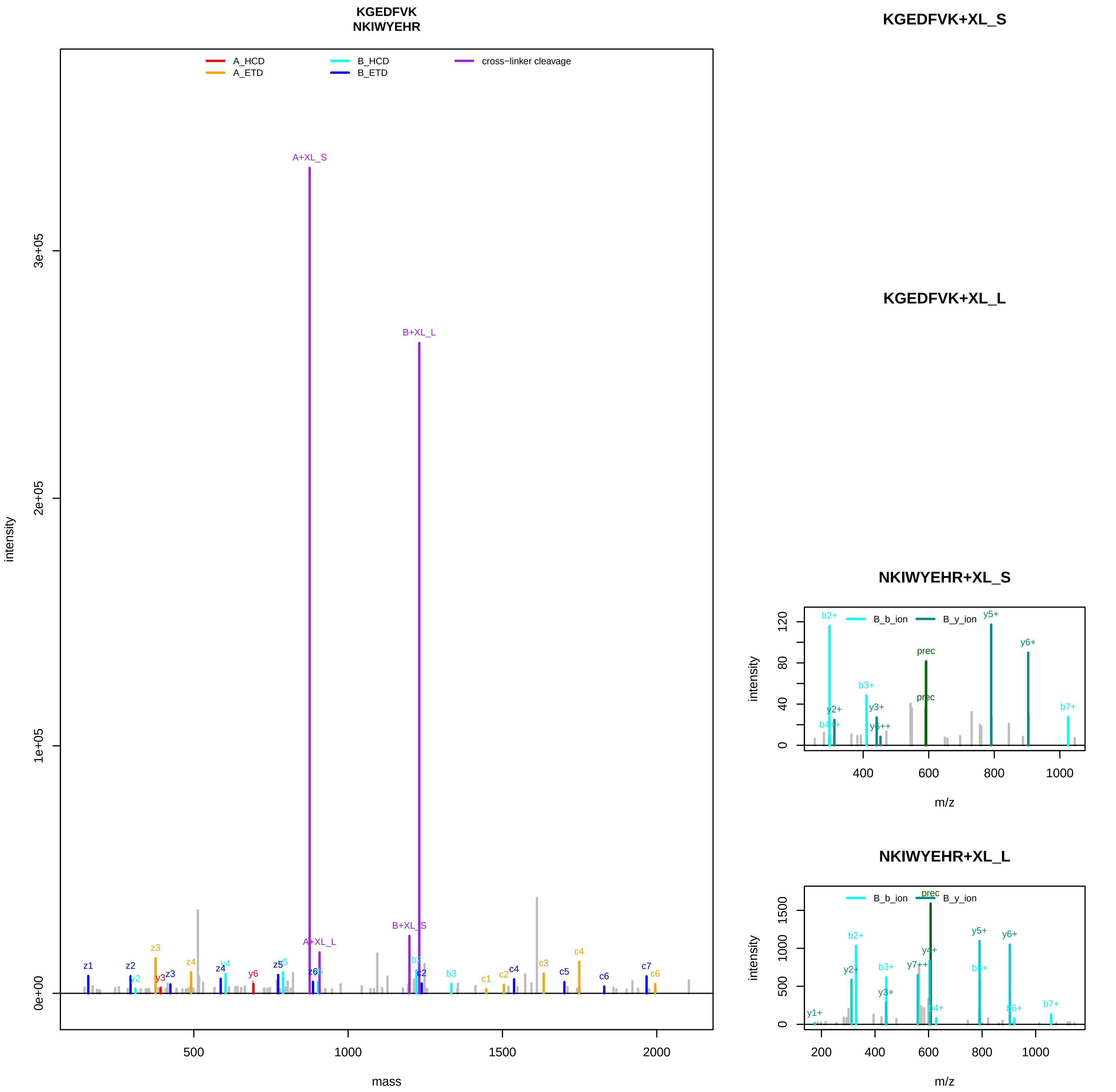

KGEDFVK
NKIWYEHR
A_HCD
A_ETD
B_HCD
B_ETD
cross−linker cleavage
intensity
mass
0e+00
1e+05
2e+05
3e+05
500
1000
1500
2000
A+XL_S
B+XL_L
B+XL_S
A+XL_L
z1
z2
y2
z3
y3
z3
z4
y4
z4
y6
z5
y5
c2
c3
c4
c5
c6
c7
c1
c2
c3
c4
c6
b2
b3
KGEDFVK+XL_S
KGEDFVK+XL_L
NKIWYEHR+XL_S
B_b_ion
B_y_ion
b2+
y5+
y6+
prec
b3+
y2+
y3+
b4++
y6++
b7+
m/z
0
40
80
120
400
600
800
1000
NKIWYEHR+XL_L
y1+
y2+
b2+
b3+
y3+
y7++
y4+
y5+
b5+
y6+
b6+
b7+
b4+
0
500
1000
1500
200
